# Association of clinic setting with quality indicator performance in systemic lupus erythematosus: a cross-sectional study

**DOI:** 10.1186/s13075-022-02823-9

**Published:** 2022-06-22

**Authors:** Sidha Sreedharan, Ning Li, Geoff Littlejohn, Russell Buchanan, Mandana Nikpour, Eric Morand, Alberta Hoi, Vera Golder

**Affiliations:** 1 Monash University, Melbourne, Australia; 2 Monash Health, Melbourne, Australia; 3 Austin Health, Melbourne, Australia; 4 The University of Melbourne at St Vincent’s Hospital Melbourne, Melbourne, Australia

**Keywords:** Quality indicators, Quality of care, Systemic lupus erythematosus

## Abstract

**Background:**

Healthcare quality for systemic lupus erythematosus (SLE) is a modifiable target for improving patient outcomes. We aimed to assess the quality of care processes in different clinic settings, comparing a subspecialty lupus clinic with hospital-based and private general rheumatology clinics.

**Methods:**

Patients with SLE (*n* = 258) were recruited in 2016 from a subspecialty lupus clinic (*n* = 147), two hospital general rheumatology clinics (*n* = 56) and two private rheumatology clinics (*n* = 55). Data were collected from medical records and patient questionnaires. Quality of care was assessed using 31 validated SLE quality indicators (QI) encompassing diagnostic work-up, disease and comorbidity assessments, drug monitoring, preventative care and reproductive health. Per-QI performance was measured as a percentage of patients that met the QI relative to the number of patients eligible. Per-patient QI performance was calculated as a percentage of QIs met relative to the number of eligible QIs for each patient. Per-QI and per-patient QI performance were compared between the three clinic settings, and multiple regression performed to adjust for sociodemographic, disease and healthcare factors.

**Results:**

Per-QI performance was generally high across all clinic settings for diagnostic work-up, comorbidity assessment, lupus nephritis, drug monitoring, prednisolone taper, osteoporosis and pregnancy care. Median [IQR] per-patient performance on eligible QIs was higher in the subspeciality lupus clinic (66.7% [57.1–74.1]) than the hospital general rheumatology (52.7% [47.5–58.1]) and private rheumatology (50.0% [42.9–60.9]) clinics (*p* <0.001) and the difference remained significant after multivariable adjustment. The subspecialty lupus clinic recorded higher per-QI performance for documentation of disease activity, disease damage, cardiovascular risk factor and drug toxicity assessments, pre-immunosuppression hepatitis and tuberculosis screening, new medication counselling, vaccinations, sun avoidance education and contraception counselling.

**Conclusions:**

SLE patients managed in a subspecialty lupus clinic recorded higher per-patient QI performance compared to hospital general rheumatology and private rheumatology clinics, in part related to better documentation on certain QIs.

**Supplementary Information:**

The online version contains supplementary material available at 10.1186/s13075-022-02823-9.

## Background

Systemic lupus erythematosus (SLE) is a chronic multisystem autoimmune disease characterised by a wide range of manifestations, from relatively mild cutaneous and musculoskeletal issues to severe end-organ disease. It is associated with significant morbidity and premature mortality [1]. Patients may be burdened by disease activity, treatment effects and long-term complications such as organ damage [2, 3], with resultant impaired health-related quality of life [4, 5]. As such, management is complex, with frequent health care interactions [6] and disparate patient outcomes.

Healthcare quality is an important potential focus for improving patient outcomes in SLE and can be assessed at different levels of healthcare using structural, process, or outcome measures [7]. Studies on structural aspects of SLE healthcare quality have largely looked at issues of access to care, and studies on outcomes of care have looked at impacts on disease activity, damage accrual and patient satisfaction [8]. Processes of care, the most commonly studied aspect of healthcare quality, refers to the interactions between healthcare professionals and patients and can be assessed using quality indicators (QI) [8]. Benchmarking performance on QIs allows for intervention with policies and pathways to ensure standardised care. There are at least two validated SLE QI sets that measure disease-specific processes of care [9, 10], and which can be utilised to identify gaps in SLE healthcare quality as areas for improvement. Higher performance on SLE QIs has been shown to be associated with lower disease activity [11] and to be protective against disease damage accrual in at least two independent cohorts [11, 12]. Delivery of high-quality care requires clear documentation [13–16] and such documentation is essential to pass certain SLE QIs.

Disease-specific subspecialty clinics have been shown to provide better quality of care in other chronic diseases such as diabetes, heart disease and certain cancers [17–19]. This is likely due to the availability of experienced subspecialists, multidisciplinary teams and efficient care pathways. A few studies have suggested superior performance of tertiary academic centres [20, 21], and more specifically, subspecialty lupus clinics in the provision of quality SLE care [22]. However, this has so far only been assessed in the United States (US), where medical insurance status is known to be a confounder of the quality of SLE care received [23–25]. Comparison of models of care may be informative under a universal healthcare system, in which access to care is not constrained by insurance.

In this study, we aimed to evaluate documented and patient-reported performance on SLE QIs in a subspecialty lupus clinic compared with hospital general rheumatology and private rheumatology clinics in a universal healthcare setting.

## Methods

### Study design and participants

In this cross-sectional study, we recruited patients in 2016 from three clinic settings in Melbourne, Australia, including a subspecialty lupus clinic (SLC), two public hospital general rheumatology clinics (HRC) and two private rheumatology practice clinics (PRC). Patients were deemed eligible if they were over 18 years old and able to consent, met American College of Rheumatology 1997 (ACR) or Systemic Lupus International Collaborating Clinics 2012 (SLICC) classification criteria for SLE [26, 27], and had at least 12 months duration of follow-up with access to electronic medical records. Eligible patients were either recruited at point-of-care at their rheumatology clinic appointment or if they returned a mailed invitation.

### Procedures and variables

Quality of care was assessed using two sets of SLE QIs from the European League Against Rheumatism (EULAR) and the US [9, 10]. Together, these 31 QIs comprehensively cover SLE care, including diagnostic work-up, disease and comorbidity assessment, drug monitoring, preventative care and reproductive health (Supplementary Table 1). All QIs and eligibility were scored nominally (yes/no) as per their defined periods via review of all electronic outpatient medical records until their recruitment (Clinical Record Form in Supplementary Table 2). Assessment of QI performance was done by an investigator external to the clinic settings. Additionally, patient self-report for amenable QIs was collected using paper questionnaires (Supplementary Table 3) either mailed or distributed to participants prior to their clinic appointment. Demographic, socioeconomic, disease and healthcare access data were gathered from either medical record review, patient questionnaire, or from the Australian Lupus Registry and Biobank (ALRB) database [28] for the SLC patients. Baseline disease activity was assessed using SLE Disease Activity Index 2000 (SLEDAI2K) [29] and irreversible organ damage using SLICC Damage Index (SDI) [30] by the principal investigator for the HRC and PRC patients and obtained from the ALRB database for the SLC patients.

### Outcomes

Per-QI performance was measured as a percentage of patients that met the QI relative to the total number of patients eligible. This was performed both for results by medical record review and patient self-report, to evaluate differences in medical documentation and patient recall. Per-patient QI performance was calculated as a percentage of QIs met relative to the total number of eligible QIs for each patient. In calculating the per-patient QI performance including patient self-report, if there was inconsistency between documentation and patient self-report and there was not a documentation requirement for a pass, then a pass on one modality was counted as a pass for that QI.

### Statistical analyses

Statistical analyses were performed using STATA version 16.1. Per-QI performance was compared between the clinic settings by the chi-square test. We performed multiple logistic regression for select QIs that were amenable to further analysis. Because some individual QIs were excluded from multiple logistic regression analysis due to small number of observations, we also calculated per-patient QI performance.

Per-patient QI performance was compared between the three clinic settings by the non-parametric Kruskal-Wallis test. We performed multiple linear regression of per-patient QI performance, adjusting for demographic (age, gender, ethnicity), socioeconomic (education, income), disease (disease duration, ACR phenotype, baseline SDI and SLEDAI2K) and healthcare (hospital insurance, family physician visits, rheumatologist visits) factors in addition to clinic setting. Regression model performance was assessed using residual versus fitted plot (Supplementary Figure 1), which showed that the model fitted the data well.

## Results

There were 258 patients recruited (147 SLC, 56 HRC and 55 PRC). Baseline characteristics according to clinic setting are outlined in Table 1. Patients from PRC were more likely to be older, Caucasian, to have private insurance and longer disease duration, when compared to SLC and HRC patients. Furthermore, they were less likely to have renal manifestations or be treated with immunosuppressants.

**Table 1:** Baseline characteristics according to clinic setting

Baseline characteristics	SLC ***N*** = 147	HRC ***N*** = 56	PRC ***N*** = 55
Age (mean (SD))	44.1 (14.3)	48.7 (14.7)	53.9 (14.3)
Female gender (*N* (%))	124 (84.4%)	50 (89.3%)	52 (94.5%)
Ethnicity			
Caucasian	82 (55.8%)	39 (69.6%)	46 (83.6%)
Asian	54 (36.7%)	14 (25.0%)	8 (14.5%)
Other	11 (7.5%)	3 (5.4%)	1 (1.8%)
Education			
Primary	14 (9.7%)	4 (7.7%)	2 (3.6%)
Secondary	49 (33.8%)	17 (32.7%)	20 (36.4%)
Tertiary	82 (56.6%)	31 (59.6%)	33 (60.0%)
Income			
<35k per annum	35 (29.7%)	12 (30.8%)	9 (20.9%)
35k–<70k per annum	47 (39.8%)	11 (28.2%)	18 (41.9%)
>70k per annum	36 (30.5%)	16 (41.0%)	16 (37.2%)
Private hospital insurance	54 (36.7%)	17 (32.7%)	37 (67.3%)
Private extras insurance	43 (29.7%)	13 (25.0%)	34 (61.8%)
Current smoker	21 (14.7%)	8 (15.4%)	3 (5.5%)
Disease duration in years (median [IQR])	10.0 (5.0–18.0)	8.0 (3.0–17.0)	17.0 (10.0–21.0)
ACR criteria^a^			
Malar rash	74 (50.3%)	16 (28.6%)	15 (27.3%)
Discoid rash	16 (10.9%)	3 (5.4%)	3 (5.5%)
Photosensitivity	59 (40.1%)	21 (37.5%)	12 (21.8%)
Oral ulcers	55 (37.4%)	10 (17.9%)	12 (21.8%)
Arthritis	107 (72.8%)	46 (82.1%)	40 (72.7%)
Serositis	58 (39.5%)	11 (19.6%)	4 (7.3%)
Renal	61 (41.5%)	18 (32.1%)	3 (5.5%)
Neurologic	13 (8.8%)	6 (10.7%)	8 (14.5%)
Haematologic	74 (50.3%)	22 (39.3%)	21 (38.2%)
Immunologic	126 (85.7%)	44 (78.6%)	36 (65.5%)
ANA	147 (100.0%)	56 (100.0%)	55 (100.0%)
SDI score (median [IQR])	1.0 (0.0–3.0)	1.0 (0.0–2.0)	1.0 (0.0–1.0)
SLEDAI score (median [IQR])	4.0 (2.0–6.0)	2.0 (0.0–4.0)	2.0 (0.0–2.0)
Medications			
Glucocorticoids	70 (47.6%)	31 (55.4%)	22 (40.0%)
Hydroxychloroquine	124 (84.4%)	45 (80.4%)	45 (81.8%)
cDMARDs	87 (59.2%)	33 (58.9%)	24 (43.6%)
bDMARDs	14 (9.5%)	4 (7.1%)	1 (1.8%)
Number of family physician visits per year			
Annually or less	31 (22.8%)	9 (17.3%)	7 (12.7%)
6 monthly	38 (27.9%)	10 (19.2%)	16 (29.1%)
3 monthly	37 (27.2%)	21 (40.4%)	11 (20.0%)
2 monthly or more	30 (22.1%)	12 (23.1%)	21 (38.2%)
Number of rheumatologist visits per year (median [IQR])	4.0 (3.0–5.0)	4.0 (2.0–4.5)	2.0 (1.0–3.0)

*Abbreviations*: *ACR* American College of Rheumatology, *ANA* Antinuclear antibody, *bDMARDs* Biologic disease modifying anti-rheumatic drugs, *cDMARDs* Conventional disease modifying anti-rheumatic drugs, HRC Hospital general rheumatology clinic, *PRC* Private rheumatology clinic, *SLC* Subspecialty lupus clinic, *SLEDAI* Systemic Lupus Erythematosus Disease Activity Index, *SDI* Systemic Lupus International Collaborating Clinics/ American College of Rheumatology Damage Index

^a^ACR criteria included to demonstrate SLE phenotype on an ‘ever-present’ basis

Median [IQR] per-patient QI performance was significantly higher in the SLC (66.7% [57.1–74.1]) than the HRC (52.7% [47.5–58.1]) and PRC (50.0% [42.9–60.9]) settings (*p* <0.001) (Table 2). This difference was also observed when the per-patient QI performance was calculated including patient self-report (SLC 73.1% [65.2–80.0] vs HRC 68.1% [60.4–71.8] vs PRC 63.2% [55.0–68.4], *p* <0.001). Sub analysis on the EULAR QIs also revealed significantly higher per-patient QI performance in the SLC compared to the HRC and PRC settings (*p* <0.001), regardless of whether medical record review or patient self-report data were used (Table 2). Per-patient performance on the US QIs by medical record review was significantly higher in the SLC than HRC or PRC settings (*p* <0.001). However, there was no statistically significant difference in per-patient US QI performance between the clinic settings when patient self-report was included (*p* = 0.34) (Table 2). After adjustment for demographic, socioeconomic, disease and healthcare access determinants, clinic setting statistically significantly predicted per-patient QI performance with coefficients (95% confidence interval) of −13.3 (−17.8, −8.9) for HRC, and −11.5 (−16.4, −6.7) for PRC, when compared to SLC (*p* <0.01) (Supplementary Table 2). Other significant variables included renal involvement (*p* <0.01), which was a positive predictor of per-patient QI performance, and disease duration (*p* <0.01) and serositis (*p* <0.05), which were negative predictors (Supplementary Table 4).

**Table 2:** Per-patient QI performance according to clinic setting

Quality indicator performance (median [IQR])	SLC (***n*** = 147)	HRC (***n*** = 56)	PRC (***n*** = 55)	***p*** value
All quality indicators				
EMR only	66.7% [57.1–74.1]	52.7% [47.5–58.1]	50.0% [42.9–60.9]	<0.001
Combined EMR and PSR	73.1% [65.2–80.0]	68.1% [60.4–71.8]	63.2% [55.0–68.4]	<0.001
EU quality indicators				
EMR only	66.7% [55.6–75.0]	45.5% [37.5–50.0]	44.4% [33.3–54.5]	<0.001
Combined EMR and PSR	66.7% [60.0–77.8]	54.5% [45.5–57.8]	44.4% [36.4–55.6]	<0.001
US quality indicators				
EMR only	66.7% [57.1–75.0]	58.3% [51.9–69.6]	61.5% [50.0–68.8]	<0.001
Combined EMR and PSR	76.9% [69.2–84.6]	80.0% [70.0–88.2]	80.0% [70.0–87.5]	0.34

*Abbreviations*: *EMR* Electronic medical record review, *EU* European, *HRC* Hospital general rheumatology clinic, *PRC* Private rheumatology clinic, *PSR* Patient self-report, *SLC* subspecialty lupus clinic, *US* United States

Performance was high (>85%) across all clinic settings for diagnostic work-up, comorbidity assessment, lupus nephritis, baseline and monitoring pathology for medications, prednisolone taper, osteoporosis and pregnancy QIs (Table 3). The PRC setting scored better at ophthalmologic screenings for patients on hydroxychloroquine (*p* = 0.003) or glucocorticoids (*p* = 0.007) while the HRC setting was better at performing disease monitoring tests (*p* <0.001) (Table 3). The SLC recorded higher performance than HRC and PRC for documentation of cardiovascular risk factor assessment (*p* <0.001), drug toxicity assessment (*p* <0.001), pre-immunosuppression hepatitis and tuberculosis screening (*p* = 0.01), new medication counselling (*p* <0.001), vaccinations (*p* <0.001), sun avoidance education (*p* = 0.01), and contraception counselling (*p* <0.001) (Table 3). Furthermore, the SLC consistently recorded validated assessments of disease activity and disease damage while neither of the other settings did so (*p* <0.001) (Table 3). None of the clinics performed validated assessments of quality of life at each visit.

**Table 3:** Per-QI performance according to clinic setting

Quality indicator	SLC *N* (%)	HRC *N* (%)	PRC *N* (%)	*p* value
Diagnostic work-up				
Suspected diagnosis work-up (US)	**50/50** **(100.0%)**	**56/56** **(100.0%)**	**55/55** **(100.0%)**	–
New diagnosis tests (US)	**50/50** **(100.0%)**	**56/56** **(100.0%)**	**50/55** **(90.9%)**	0.007
Autoantibodies at diagnosis (EU)	**50/50** **(100.0%)**	**56/56** **(100.0%)**	**54/55** **(98.2%)**	0.38
Disease and comorbidities assessment				
Assessment of disease activity at each visit (EU)	**147/147** **(100.0%)**	0/56 (0.0%)	0 /55 (0.0%)	<0.001
Assessment of disease damage at each visit (EU)	**141/147** **(95.9%)**	0/56 (0.0%)	0/55 (0.0%)	<0.001
Evaluation of quality of life at each visit (EU)	0/147 (0.0%)	0/56 (0.0%)	0/55 (0.0%)	–
Record comorbidities at least once a year (EU)	**147/147** **(100.0%)**	**52/56** **(92.9%)**	**50/55** **(90.9%)**	0.002
Monitoring tests every six months (EU)	**132/147** **(89.8%)**	**54/56** **(96.4%)**	22/55 (40.0%)	<0.001
Assessment of cardiovascular risk factors (US)	91/147 (61.9%)	14/56 (25.0%)	8/55 (14.6%)	<0.001
Three monthly tests if evidence of renal disease (US)	**43/44** **(97.7%)**	**15/16** **(93.8%)**	**2/2** **(100.0%)**	0.50
Treatment within one month of diagnosis of proliferative lupus nephritis (US)	**43/43** **(100.0%)**	**12/12** **(100.0%)**	**3/3** **(100.0%)**	–
Anti-hypertensive treatment in lupus renal disease (US)	**22/22** **(100.0%)**	**5/5** **(100.0%)**	–	–
ACEI/ARB treatment if proteinuria (US)	**42/44** **(95.5%)**	7/10 (70.0%)	–	0.01
Medications				
Assessment for drug toxicity at each visit (EU)	**141/147** **(95.9%)**	43/56 (76.8%)	44/54 (81.5%)	<0.001
Ophthalmologic review if on hydroxychloroquine as per guidelines (EU)	46/103 (44.7%)	11/34 (32.4%)	27/38 (71.1%)	0.003
Ophthalmologic review if on glucocorticoids as per guidelines (EU)	34/67 (50.8%)	5/22 (22.7%)	16/23 (69.6%)	0.007
Hepatitis B and C and tuberculosis testing prior to starting immunosuppression (EU)	59/95 (62.1%)	19/41 (46.3%)	9/28 (32.1%)	0.01
Counselling when prescribed new medications (US)	118/139 (84.9%)	14/48 (29.2%)	26/44 (59.1%)	<0.001
Baseline studies when prescribed new medications (US)	**139/139** **(100.0%)**	**45/45** **(100.0%)**	**44/44** **(100.0%)**	–
Monitoring tests for established medications (US)	**143/145** **(98.6%)**	**51/51** **(100.0%)**	**50/52** **(96.1%)**	0.28
Attempt to taper prednisolone if >10 mg for >3 months (US)	**75/75** **(100.0%)**	**22/22** **(100.0%)**	**15/15** **(100.0%)**	–
Bone mineral density testing if received prednisolone >7.5 mg for >3 months (US)	**100/106** **(94.3%)**	**30/31** **(96.8%)**	18/23 (78.3%)	0.02
Calcium and vitamin D if received prednisolone >7.5 mg for >3 months (US)	**93/105** **(88.6%)**	**27/31** **(87.1%)**	**22/24** **(91.7%)**	0.86
Osteoporosis treatment (US)	**18/20** **(90.0%)**	**11/12** **(91.7%)**	**9/9** **(100.0%)**	0.63
Preventative care				
All patients should be vaccinated against influenza and pneumococcus (EU)	34/147 (23.1%)	1/55 (1.8%)	2/55 (3.6%)	<0.001
Influenza vaccination if on immunosuppressants (US)	64/96 (66.7%)	5/46 (10.9%)	2/36 (5.6%)	<0.001
Pneumococcal vaccination if on immunosuppressants (US)	21/96 (21.9%)	1/46 (2.2%)	1/34 (2.9%)	<0.001
Sun avoidance counselling ever (US)	27/147 (18.4%)	5/56 (8.9%)	2/55 (3.6%)	0.01
Reproductive health				
Ro, La and antiphospholipid antibody testing in pregnancy (US)	**14/14** **(100.0%)**	**6/6** **(100.0%)**	**5/5** **(100.0%)**	–
Aspirin and heparin offered for subsequent pregnancies if antiphospholipid syndrome pregnancy complications (US)	**2/2** **(100.0%)**	**2/2** **(100.0%)**	**2/2** **(100.0%)**	–
Teratogenic medication risk and contraception counselling in reproductive age women (US)	42/64 (65.6%)	4/20 (20.0%)	6/14 (42.9%)	0.001

Bold: high performance with pass rate >85% of eligible patients

*Abbreviations*: *EU* European, *HRC* Hospital general rheumatology clinic, *PRC* Private rheumatology clinic, *SLC* Subspecialty lupus clinic, *US* United States

There was higher per-QI performance via patient self-report than via medical record review for the following QIs: hydroxychloroquine and glucocorticoid ophthalmologic screening, pneumococcal vaccination, sun avoidance counselling and contraception counselling (Table 4). In contrast, there was higher QI performance via medical record review than patient self-report for drug toxicity assessment. Patient self-report and medical record review results were similar for the calcium and vitamin D supplementation QI.

**Table 4:** Comparison of documented performance and patient self-report according to clinic setting

Quality indicator	SLC		HRC		PRC	
	EMR	PSR	EMR	PSR	EMR	PSR
*Evaluation of quality of life (EU)*	0/147 (0.0%)	27/140 (19.3%)	0/56 (0.0%)	15/52 (28.9%)	0/55 (0.0%)	18/55 (32.7%)
Assessment of cardiovascular risk factors by rheumatologist (US)	91/147** (61.9%)		14/56** (25.0%)		8/55** (14.5%)	
*Assessment of cardiovascular risk factors by family physician*		50/139* (36.0%)		21/51* (41.2%)		31/54* (57.4%)
*Assessment of cardiovascular risk factors by either rheumatologist or family physician*	107/144* (74.3%)		30/53* (56.6%)		32/54* (59.3%)	
*Assessment for drug toxicity (EU)*	141/147** **(95.9%)**	37/138 (26.8%)	43/56** (76.8%)	23/52 (44.2%)	44/54** (81.5%)	20/54 (37.0%)
Ophthalmologic review if on hydroxychloroquine as per guidelines (EU)	46/103** (44.7%)	69/103 (67.0%)	11/34** (32.4%)	24/31 (77.4%)	27/38** (71.1%)	**33/38 (86.8%)**
Ophthalmologic review if on glucocorticoids as per guidelines (EU)	34/67** (50.8%)	45/67 (67.2%)	5/22** (22.7%)	**18/21 (85.7%)**	16/23** (69.6%)	**20/23 (86.9%)**
Counselling when prescribed new medications (US)	118/139** (84.9%)	116/144 (80.6%)	14/48** (29.2%)	**44/47 (93.6%)**	26/44** (59.1%)	**38/44 (86.4%)**
Calcium and vitamin D supplementation if received prednisolone >7.5 mg for >3 months (US)	**93/105 (88.6%)**	**94/104 (90.4%)**	**27/31 (87.1%)**	**29/29 (100%)**	**22/24 (91.7%)**	**22/24 (91.7%)**
All patients should be vaccinated against influenza and pneumococcus (EU)	34/147** (23.1%)	27/139 (19.4%)	1/55** (1.8%)	16/50 (32.0%)	2/55** (3.6%)	8/54 (14.8%)
*All patients should be vaccinated against influenza*	–	92/147 (62.6%)	–	34/52 (65.4%)	–	36/55 (65.5%)
*All patients should be vaccinated against pneumococcus*	–	27/139 (19.4%)	–	16/50 (32.0%)	–	9/54 (16.7%)
*Rheumatologist recommended vaccination?*	–	82/136* (60.3%)	–	28/52* (53.9%)	–	20/55* (36.4%)
Influenza vaccination if on immunosuppressants (US)	64/96** (66.7%)	61/96 (63.5%)	5/46** (10.9%)	30/43 (69.8%)	2/36** (5.6%)	24/36 (66.7%)
Pneumococcal vaccination if on immunosuppressants (US)	21/96** (21.9%)	24/91 (26.4%)	1/46** (2.2%)	16/41 (39.0%)	1/34** (2.9%)	8/33 (24.2%)
Sun avoidance counselling ever (US)	27/147* (18.4%)	**125/144 (86.8%)**	5/56* (8.9%)	**45/52 (86.5%)**	2/55* (3.6%)	**47/55 (85.5%)**
Teratogenic medication risk and contraception counselling in reproductive age women (US)	42/64** (65.6%)	53/63** (84.1%)	4/20** (20.0%)	9/17** (52.9%)	6/14** (42.9%)	5/13** (38.5%)

Italics: PSR questions that did not match the quality indicator statement exactly as were used to gain additional information and were not used in the per-patient quality indicator performance calculation

Note: The differences in the numbers of eligible patients between EMR and PSR are due to missing values if predating EMR or if unanswered by PSR

*Abbreviations*: *EMR* Electronic medical record review, *EU* European, *HRC* Hospital general rheumatology clinic, *PRC* Private rheumatology clinic, *PSR* Patient self-report, *SLC* subspecialty lupus clinic, *US* United States

*p<0.05, **p<0.01

After adjusting for demographic, socioeconomic, disease and healthcare access variables, when compared to SLC, patients in the HRC were less likely to receive documented care consistent with the following QIs: cardiovascular risk factor assessment (OR 0.18 (0.06, 0.48), *p* <0.01), new medication counselling (OR 0.03 (0.01, 0.12), *p* <0.01), sun avoidance counselling (OR 0.07 (0.01, 0.75), *p* <0.05) and contraceptive counselling (OR 0.06 (0.01, 0.44), *p* <0.01). Patients in the PRC were less likely to receive documented care consistent with the following QIs when compared to SLC: disease monitoring tests (OR 0.06 (0.01, 0.31), *p* <0.01) and cardiovascular risk factor assessment (OR 0.16 (0.05, 0.52), *p* <0.01) (Supplementary Table 5).

## Discussion

Provision of high-quality care holds the potential to improve patient outcomes in the absence of breakthrough new therapies for SLE, which have been elusive for non-renal lupus. However, this is challenging because of the complex and heterogeneous nature of disease manifestations and clinical course. The driving force for any quality measurement programme is to identify gaps and thereby derive strategies to improve care. In this cross-sectional study, we have benchmarked the performance of lupus QIs across different care settings, to determine whether clinic type impacted on quality of care.

We observed that the SLC recorded higher per-patient QI performance compared to the other clinic settings. The SLC studied is the founding site for the Australian Lupus Registry and Biobank [28] and has several routine processes in place that contribute to higher documented QI performance. At the SLC, it is a requirement that disease activity is assessed and documented at every visit using SLEDAI-2K and disease damage assessed annually using SDI, explaining the high pass rates for these QIs. Other measures that may explain higher QI performance include the use of pre-printed laboratory test orders and electronic medical record prompts for routine preventive care. Although these processes are inherent to a research clinic, they nonetheless contribute to the overall quality of care provided. The HRC setting involves tertiary clinics that service a range of rheumatological conditions, and the PRC setting includes private practice clinicians in shared care with family physicians and other specialists. There are elements of SLE care that may be primarily managed by the family physician and therefore not documented by the rheumatologist, such as preventative health QIs related to vaccinations and cardiovascular screening. We attempted to capture this in our study by including patient self-report for amenable QIs. For example, the cardiovascular risk factor assessment QI was assessed by the patients’ rheumatologist entry in the medical record or by the family physician as captured on patient survey.

Our study highlights the importance of documentation within the medical record to QI performance. We measured per-QI performance via both medical record review and patient self-report for amenable QIs. Unlike in a previous study [22], we reported these results separately rather than in combination, as there are some QIs that require documentation, thus making patient recall alone insufficient for a pass result. In a complex disease like SLE, with multiple healthcare providers involved, documentation is essential to ensure safe care [31]. The higher per-patient and per-QI performance for ophthalmologic screening, pneumococcal vaccination, sun avoidance and contraception counselling when including patient self-report compared to medical record review alone highlights the gaps in documentation across clinic settings. However, patient self-report is also likely more important when assessing QIs related to patient understanding, for example, sun avoidance. Interestingly, performance on the drug toxicity QI was higher via medical record review compared to patient self-report, which may be related to patient recall or understanding of drug toxicity assessment.

We found that in addition to clinic setting, clinical characteristics such as disease duration, serositis and renal disease were significant factors impacting on per-patient QI performance. Better QI performance in patients with renal involvement potentially reflects more severe disease prompting clinicians to provide more comprehensive care. In contrast, and unlike in studies from the US [23, 25, 32], socioeconomic factors like medical insurance status, income and education level were not significantly associated with per-patient QI performance in the universal healthcare setting in which this study was performed. Accordingly, predictors of poorer outcomes in SLE like medical insurance status, income, education level and ethnicity reported in US studies [33] did not have a significant impact on the per-patient QI performance. We found no statistically significant association of per-patient QI performance with ethnicity. Although the Australian population has a different ethnicity mix compared with the US, it is ethnically diverse with a large number of patients of Asian ancestry [34]. Asians, like African Americans, have more severe SLE [35], and therefore our population may reflect a similar spectrum of disease to that reported in US studies.

In this study, we chose to include two QI sets as they differ in the way quality of care is measured. The differences in per-patient QI performance between the clinic settings were more marked using the EULAR QI set compared with the US QI set. This is likely because the EULAR QI set has three QIs related to use of validated formal assessment tools [29, 30, 36], all of which were consistently performed in the SLC but not the other settings; clinical assessments of disease made in the HRC and PRC settings, for example, documentation of ‘active arthritis’, were not sufficient for a pass as defined by the QIs. Evaluation of quality of life was passed if recorded formally at every visit for medical record review or if the patient reported via questionnaire that their quality of life was assessed. Health-related quality of life is assessed annually at the SLC; however, the EULAR QI for this required it to be performed at each visit, resulting in a 0% pass rate per medical record review. Even though none of the clinics assessed quality of life formally at every visit, some patients felt that this QI was addressed in their consultation. The feasibility of implementing instrument-based health-related quality of life measures at every visit is uncertain.

The findings of this study revealed that performance across all clinic settings in some domains, for example, osteoporosis and lupus nephritis QIs, were much higher than previously reported [37–39]. Some key areas of lower performance included influenza vaccination, cardiovascular risk assessment and contraceptive counselling QIs, although performance in our study were either similar or higher than that reported in other cohorts [32, 39, 40]. Performance on the pneumococcal vaccination QI was much lower than that previously reported [32] across all clinic settings. A previous study found that failure of healthcare provider recommendation was the most common reason why SLE patients did not receive influenza and pneumococcal vaccines, suggesting potential benefit of intervention at the provider level [41]. We found a statistically significant difference across the clinic settings for cardiovascular risk factor assessment even when including screening by the family physician, highlighting another area for improvement, as clearer communication of responsibility and results between the healthcare providers may facilitate this. The use of clinical record prompts, as implemented in the SLC, may improve performance on these preventative health QIs.

Limitations of this study include the small number of observations for some QIs, for example the pregnancy-related QIs. As it was a cross-sectional study, we did not assess QI performance trends over time or impact on patient outcomes. We included patient self-report for amenable QIs, introducing an element of recall bias. There might also be inherent subjectivity in physician care provision for varying disease severity. Strengths of the current study include that it compared settings within a universal healthcare system, reducing the impact of socioeconomic and insurance status on the results, utilised both EULAR and US QIs, and assessments of QI performance were done by an investigator external to the clinic settings.

## Conclusions

Review of QI performance is an important aspect of improving quality of care, by providing a benchmark for care delivery and the opportunity to identify gaps to address. This study highlights that quality of care is measurable, and different, across clinic settings that care for SLE patients in a universal healthcare system. We found that for multiple QIs SLE patients across all clinic settings received care consistent with the standards. However, SLE patients managed in a subspecialty lupus clinic recorded higher per-patient QI performance, compared to hospital and private general rheumatology clinics. Importantly, we did not find that socioeconomic factors, insurance status or ethnicity, implicated in lower quality of care in studies in the US, predicted per-patient QI performance in a universal healthcare setting. Strategies to improve QI performance through documentation, education and other measures, and analysis of the relationship between improved QI performance and disease- and patient-reported outcomes, should be further evaluated.

## Supplementary Information

**Additional file 1: Supplementary Table 1.** List of all quality indicators.

**Additional file 2: Supplementary Table 2.** Clinical record form.

**Additional file 3: Supplementary Table 3.** Patient questionnaire.

**Additional file 4: Supplementary Table 4.** Multivariable regression of per-patient QI performance adjusted for sociodemographic, disease and healthcare determinants.

**Additional file 5: Supplementary Table 5.** Multivariable regression of per-QI performance adjusted for sociodemographic, disease and healthcare determinants.

**Additional file 6: Supplementary Figure 1.** Per-patient QI performance linear multivariable regression model residual versus fit plot.

## Data Availability

The dataset generated and analysed during the current study is the property of Monash University and is available from the corresponding author on reasonable request.

## Abbreviations

ACR: American College of Rheumatology
ALRB: Australian Lupus Registry Biobank
EMR: Electronic medical record review
EULAR: European League Against Rheumatism
HRC: Hospital general rheumatology clinic
IQR: Interquartile range
PRC: Private rheumatology clinic
PSR: Patient self-report
QI: Quality indicator
SDI: Systemic Lupus International Collaborating Clinics/American College of Rheumatology Damage Index
SLC: Subspecialty lupus clinic
SLE: Systemic lupus erythematosus
SLEDAI2K: Systemic Lupus Erythematosus Disease Activity Index 2000
SLICC: Systemic Lupus International Collaborating Clinics
US: United States
