# Supplementary material for: Association of clinic setting with quality indicator performance in systemic lupus erythematosus: a cross-sectional study
Source: Arthritis Res Ther. 2022 Jun 22;24:150. doi: 10.1186/s13075-022-02823-9 (PMC9214991; doi:10.1186/s13075-022-02823-9)
Supplement: Supplementary file 1 — Additional file 1: Supplementary Table 1. List of all quality indicators. [file 13075_2022_2823_MOESM1_ESM.pdf]

Supplementary Table 1: List of all quality indicators

EU Quality Indicators
IF a patient is diagnosed with SLE, THEN the treating physician should assess and record disease activity using a validated activity index at each visit.
IF a patient is diagnosed with SLE, THEN the treating physician should assess and record disease damage by the SLICC/ACR Damage Index annually.
IF a patient is diagnosed with SLE, THEN he/she should provide an evaluation of his/her quality of life at each visit – either on a visual analogue scale from 0 to 10 or using any validated index (SF-36, SLE-QoL).
IF a patient is diagnosed with SLE, THEN the treating physician should assess the presence of drug toxicity at each visit and record the data in the clinical chart. Alternatively, the physician should record the absence of drug toxicity.
IF a patient is diagnosed with SLE, THEN the treating physician or a specialized nurse should record the presence of comorbid conditions at each visit. Absence of comorbidities should also be recorded.
If a patient is diagnosed with SLE and treated with hydroxychloroquine/chloroquine THEN he/she should undergo an ophthalmologic assessment according with the existing guidelines, and this should be documented in the clinical chart.
If a patient is diagnosed with SLE and treated with corticosteroids, THEN he/she should undergo an ophthalmologic assessment for the presence of cataracts and/or glaucoma according with the existing guidelines. This should be documented in the clinical chart.
IF a patient is diagnosed with SLE THEN at least every six months the rheumatologist should request the following laboratory assessment: complete blood count, erythrocyte sedimentation rate, albumin, serum creatinine or eGFR, urinalysis and protein/creatinine ratio (or 24hour proteinuria), C3 and C4.
If a patient is diagnosed with SLE and is prescribed high dose corticosteroids and/or immunosuppressive drugs THEN, based on patient's history, the rheumatologist should consider the evaluation of Hepatitis B, Hepatitis C and tuberculosis screening and record the results into the clinical chart before starting therapy.
If a patient is diagnosed with SLE, THEN the patient's history of vaccinations should be documented. Patients should be vaccinated against influenza and pneumococcus (preferably without adjuvant), if there are no contraindication to immunization.
If a patient is diagnosed with SLE, THEN the following autoantibodies should be evaluated at the first visit: ANA, anti-dsDNA, anti-Ro, anti-La, anti-RNP, anti-Sm, anti-phospholipid antibodies.
US Quality Indicators
IF a patient has a suspected diagnosis of SLE THEN an initial work-up should include the following: ANA, CBC with differentials, platelet count, serum creatinine, and urinalysis.
IF a patient is newly diagnosed with SLE THEN the following laboratory tests should be ordered within 6 months of diagnosis: anti-dsDNA, complement levels, and antiphospholipid antibodies.
IF a patient has SLE THEN education about sun avoidance should be documented at least once in the medical record (e.g., wearing protective clothing, applying sunscreens whenever outdoors, and avoiding sunbathing).
IF a patient with SLE is on immunosuppressive therapy THEN an inactivated influenza vaccination should be administered annually, unless patient refusal or contraindications are noted.
IF a patient with SLE is on immunosuppressive therapy THEN a pneumococcal vaccine should be administered, unless patient refusal or contraindications are noted.
IF a patient with SLE has received prednisone (or other glucocorticoid equivalent) $\geq$7.5 mg/ day for $\geq$3 months THEN the patient should have BMD testing documented in the medical record (either within the 12 months preceding or the 6 months after initiation), unless the patient is currently receiving antiresorptive or anabolic therapy.
IF a patient with SLE has received prednisone (or other glucocorticoid equivalent) $\geq$7.5 mg/ day for $\geq$3 months THEN supplemental calcium and vitamin D should be prescribed or recommended and documented.
IF a patient with SLE has received prednisone (or other glucocorticoid equivalent) $\geq$7.5 mg/ day for $\geq$1month and has a central T score less than or equal to $\geq$2.5 or a history of fragility fracture THEN the patient should be treated with an antiresorptive or anabolic agent, unless patient refusal or contraindications are noted.
IF a patient is prescribed a new medication for SLE (e.g., NSAIDs, DMARDs, or glucocorticoids) THEN a discussion with the patient about the risks versus benefits of the chosen therapy should be documented.
IF a patient with SLE is newly prescribed an NSAID, DMARD, or glucocorticoid THEN baseline studies should be documented within an appropriate period of time from the original prescription.
IF a patient with SLE has established treatment with an NSAID, DMARD, or glucocorticoid THEN monitoring for drug toxicity should be performed.

IF a patient with SLE is taking prednisone (or other steroid equivalent) $\geq$10 mg for $\geq$3months THEN an attempt should be made to taper the prednisone, add a steroid-sparing agent, or escalate the dose of an existing steroid-sparing agent, unless patient refusal or contraindications are noted.
IF a patient has had evidence of SLE renal disease (increasing proteinuria, active urinary sediment, a rising creatinine level, or disease activity on renal biopsy) in the past 2 years THEN the following should be obtained at 3 monthly intervals: CBC, serum creatinine, urinalysis with microscopic evaluation, and measurement of urine protein using a quantitative assay.
IF a patient is diagnosed with proliferative SLE nephritis (WHO or ISN/RPS class III or IV) THEN therapy with corticosteroids combined with another immunosuppressant agent should be provided and documented within 1 month of this diagnosis, unless patient refusal or contraindications are noted.
IF a patient with SLE has renal disease (proteinuria $\geq$300 mg/day or eGFR $\geq$60 ml/minute) and $\geq$2 BP readings, including the last reading, with systolic BP $\geq$130 mm Hg or diastolic BP $\geq$80 mm Hg over 3 months THEN pharmacologic therapy for hypertension should be initiated or the current regimen should be changed or escalated, unless patient refusal or contraindications are noted.
IF a patient with SLE has proteinuria $\geq$300 mg/day THEN the patient should be treated with an ACE inhibitor or ARB, unless patient refusal or contraindications are noted.
IF a patient has SLE THEN risk factors for cardiovascular disease, including smoking status, BP, BMI, diabetes, and serum lipids (including total cholesterol, HDL, LDL, and triglycerides), should be evaluated annually.
IF a patient with SLE is pregnant THEN anti-SSA, anti-SSB, and antiphospholipid antibodies should be documented in the medical record.
IF a patient has had pregnancy complications as a result of antiphospholipid syndrome THEN the patient should be offered aspirin and heparin (i.e., heparin or low molecular weight heparin) during subsequent pregnancies.
IF a woman between 18 and 45 years of age is started on any of the following medications for SLE: chloroquine, quinacrine, methotrexate, azathioprine, leflunomide, mycophenolate mofetil, cyclosporine, cyclophosphamide, or thalidomide THEN a discussion with the patient about the potential teratogenic risks of therapy and about contraception should be documented prior to drug initiation, unless the patient is unable to conceive (e.g., has had a hysterectomy, oophorectomy, tubal ligation, or is postmenopausal).

Abbreviations: ACE inhibitor angiotensin-converting enzyme inhibitor, ANA antinuclear antibody, anti-dsDNA anti–double-stranded DNA, ARB angiotensin receptor blocker, BMD bone mineral density, BMI body mass index, BP blood pressure, CBC complete blood cell count, DMARD disease-modifying antirheumatic drug, eGFR estimated glomerular filtration rate, EU European, HDL high-density lipoprotein, ISN/RPS International Society of Nephrology/Renal Pathology Society, LDL low-density lipoprotein, NSAID nonsteroidal anti-inflammatory drug, SF-36 36-Item Short Form Survey, SLE systemic lupus erythematosus, SLE-QoL Systemic Lupus Erythematosus Quality of Life Questionnaire, SLICC/ACR Systemic Lupus International Collaborating Clinics, WHO World Health Organization, US United States
