# Supplementary material for: Association of clinic setting with quality indicator performance in systemic lupus erythematosus: a cross-sectional study
Source: Arthritis Res Ther. 2022 Jun 22;24:150. doi: 10.1186/s13075-022-02823-9 (PMC9214991; doi:10.1186/s13075-022-02823-9)
Supplement: Supplementary file 2 — Additional file 2: Supplementary Table 2. Clinical record form. [file 13075_2022_2823_MOESM2_ESM.pdf]

Supplementary Table 2: Clinical record form

Patient ID:		
Baseline and Demographic Data	Format	
Visit Date	[dd/mm/yyyy]	
Date of Birth	[dd/mm/yyyy]	
Gender	[M/F]	
Ethnicity		
Healthcare Characteristics		
Health care setting	1(SLC)/ 2(HRC)/ 3(PRC)	
Number of rheumatology visits in the last year		
Regular GP (PSR)	[Y/N]	
Number of GP visits in the last year (PSR)		
Socioeconomic		
Education level (PSR)	[Primary/ Secondary/ Tertiary]	
Household income (PSR)		
Postcode		
Hospital insurance (PSR)	[Y/N]	
Extras insurance (PSR)	[Y/N]	
Disease		
Year of onset SLE symptoms	[yyyy]	
Year of SLE diagnosis	[yyyy]	
Smoking status (PSR)	Current/ Ex/ Non-	
Family history of SLE (PSR)	[Y/N]	
ACR criteria (Y/N)		
Malar rash	[Y/N]	
Discoid rash	[Y/N]	
Photosensitivity	[Y/N]	
Oral ulcers	[Y/N]	
Arthritis	[Y/N]	
Serositis	[Y/N]	
Renal disorder	[Y/N]	
Neurologic disorder	[Y/N]	
Haematologic disorder	[Y/N]	
Immunological disorders	[Y/N]	
Antinuclear antibody	[Y/N]	
Fulfilled at least 4 of 11 criteria	[Y/N]	
Number of ACR criteria		
SLICC Classification Criteria		
Clinical Criteria		
1. Acute cutaneous lupus, including:	[Y/N]	
Lupus malar rash		
Bullous lupus		
Toxic epidermal necrolysis		
Maculopapular lupus rash		
Photosensitive lupus rash		
Subacute cutaneous lupus		
2. Chronic cutaneous lupus, including:	[Y/N]	
Classic discoid rash		
Localized (above the neck)		
Generalized		
Hypertrophic (verrucous) lupus		
Lupus panniculitis (profundus)		
Mucosal lupus		
Lupus erythematosus tumidus		
Chilblains lupus		
Discoid lupus/lichen planus overlap		
3. Oral/nasal ulcers	[Y/N]	
4. Nonscarring alopecia	[Y/N]	
5. Synovitis ≥ 2 or	[Y/N]	
Tenderness ≥ 2 joints & 30mins of early morning stiffness		
6. Serositis, including:	[Y/N]	
Pleuritic pain		
Pleural effusions		
Pleural rub		
Typical pericardial pain		
Pericardial effusion		
Pericardial rub		
Pericarditis by electrocardiogram		

7. Renal:	[Y/N]	
Urine >500 mg protein/24 hours		
Red blood cell casts		
8. Neurologic:	[Y/N]	
Seizures		
Psychosis		
Mononeuritis multiplex		
Myelitis		
Peripheral or cranial neuropathy		
Acute confusional state		
9. Haemolytic anaemia:	[Y/N]	
10. Leukopenia <4,000/mm^3, or	[Y/N]	
Lymphopenia <1,000/mm^3		
11. Platelets <100,000/mm^3	[Y/N]	
Immunologic criteria		
1. ANA level above laboratory reference range	[Y/N]	
2. Anti-dsDNA	[Y/N]	
3. Anti-Sm	[Y/N]	
4. Anti-phospholipid antibodies:	[Y/N]	
Lupus anticoagulant		
Anti-cardiolipin		
False-positive RPR		
Beta2glycoprotein		
5. Low complement:	[Y/N]	
Low C3		
Low C4		
Low CH50		
6. Direct Coombs' test in the absence of haemolytic anaemia	[Y/N]	
SLICC: >4 criteria (at least 1 clinical & 1 immunological)		
SLICC: Nephritis + 1 immunological criteria	[Y/N]	
Pathology		
Most recent collection date:	[dd/mm/yyyy]	
General Biochemistry		
Creatinine		
eGFR	ml/min	
Albumin	g/L	
CRP		
C3	g/L	
C4	g/L	
Urine Biochemistry		
Urine protein/ creatinine ratio	g/mmol	
General Haematology		
Haemoglobin	g/L	
White cell count	x10^9/L	
Platelet	x10^9/L	
Neutrophils	x10^9/L	
Lymphocytes	x10^9/L	
ESR	mm/hr	
Immunology		
anti-dsDNA result		
Normal range of anti-dsDNA assay		
Urine MCS		
White cell count (pyuria)	x10^6/L	
Red cell count	x10^6/L	
Annual pathology date:	[dd/mm/yyyy]	
Cholesterol		
Blood glucose level		
Prior to commencing therapy:		
Quantiferon Gold	Y/N	
Hepatitis B serology	Y/N	
Hepatitis C serology	Y/N	
Current Medications		
Prednisolone (or equivalent)	mg/day	
Hydroxychloroquine	mg/day	
Methotrexate	mg/week	
Azathioprine	mg/day	
Mycophenolate mofetil	mg/day	
Mycophenolic acid	mg/day	
Leflunomide	mg/day	
Cyclosporin	mg/day	
Tacrolimus	mg/day	

Cyclophosphamide – Preceding 6 months	Y/N	
Rituximab – Preceding 6 months	Y/N	
Belimumab	Y/N	
Current SLICC		
Any cataract ever	0/1	
Retinal change or optic atrophy	0/1	
Cognitive impairment/major psychosis	0/1	
Seizures	0/1	
Stroke	0/1/2	
Cranial or peripheral neuropathy	0/1	
Transverse myelitis	0/1	
eGFR<50%	0/1	
Proteinuria >3.5gm/24hours	0/1	
End-stage renal disease	0/1	
Pulmonary hypertension	0/1	
Pulmonary fibrosis	0/1	
Shrinking lung (radiograph)	0/1	
Pleural fibrosis (radiograph)	0/1	
Pulmonary infarction (radiograph)	0/1	
Angina or coronary artery bypass	0/1	
Myocardial infarction ever	0/1/2	
Cardiomyopathy	0/1	
Valvular disease	0/1	
Pericarditis or pericardiectomy	0/1	
Claudication for 6 months	0/1	
Minor tissue loss (pulp space)	0/1	
Significant tissue loss ever	0/1/2	
Venous thrombosis or venous stasis	0/1	
Infarction or resection of bowel	0/1/2	
Mesenteric insufficiency	0/1	
Chronic peritonitis	0/1	
Stricture or upper GI surgery ever	0/1	
Muscle atrophy or weakness	0/1	
Deforming or erosive arthritis	0/1	
Osteoporosis	0/1	
Avascular necrosis (score 2 if >1)	0/1/2	
Osteomyelitis	0/1	
Scarring chronic alopecia	0/1	
Extensive scarring or panniculum	0/1	
Skin ulceration for >6 months	0/1	
Premature gonadal failure	0/1	
Diabetes (regardless of treatment)	0/1	
Malignancy	0/1	
SLICC Score		
Current SLEDAI	Score	
Seizure	0/8	
Psychosis	0/8	
Organic Brain Syndrome	0/8	
Visual Disturbance	0/8	
Cranial Nerve Disorder	0/8	
Lupus Headache	0/8	
Stroke	0/8	
Vasculitis	0/8	
Arthritis	0/4	
Myositis	0/4	
Urinary Casts	0/4	
Haematuria	0/4	
Proteinuria	0/4	
Pyuria	0/4	
Rash	0/2	
Alopecia	0/2	
Mucosal Ulcers	0/2	
Pleurisy	0/2	
Pericarditis	0/2	
Low Complement	0/2	
Increased DNA binding	0/2	
Fever	0/1	
Thrombocytopaenia	0/1	
Leucopaenia	0/1	
SLEDAI Score (max 105)		

Quality indicators - EU set		
IF a patient is diagnosed with SLE, THEN the treating physician should assess and record disease activity using a validated index at each visit.	Eligible Y/N	
	Passed Y/N/NA	
IF a patient is diagnosed with SLE, THEN the treating physician should assess and record disease damage by the SDI annually.	Eligible Y/N	
	Passed Y/N/NA	
If a patient is diagnosed with SLE, THEN he/she should provide an evaluation of his/her quality of life at each visit.	Eligible Y/N	
	Passed on EMR Y/N/NA	
	Passed on PSR Y/N/NA	
IF a patient is diagnosed with SLE, THEN the treating physician should assess the presence of drug toxicity at each visit and record the data in the clinical chart. Alternatively, the physician should record the absence of drug toxicity.	Eligible Y/N	
	Passed on EMR Y/N/NA	
	Passed on PSR Y/N/NA	
IF a patient is diagnosed with SLE, THEN the treating physician or a specialised nurse should record the presence of comorbidities at each visit.	Eligible Y/N	
	Passed Y/N/NA	
IF a patient is diagnosed with SLE and treated with hydroxychloroquine, THEN he/she should undergo an ophthalmologic assessment according to current guidelines, and this should be recorded.	Eligible Y/N	
	Passed on EMR Y/N/NA	
	Passed on PSR Y/N/NA	
IF a patient is diagnosed with SLE and treated with corticosteroids, THEN he/she should undergo an ophthalmologic assessment for cataracts according to current guidelines, and this should be recorded.	Eligible Y/N	
	Passed on EMR Y/N/NA	
	Passed on PSR Y/N/NA	
IF a patient is diagnosed with SLE, THEN at least every six months the rheumatologist should request the following: CBC, ESR, albumin, creatinine or eGFR, urinalysis and urine PCR, C3 and C4.	Eligible Y/N	
	Passed Y/N/NA	
IF a patient is diagnosed with SLE and prescribed high dose corticosteroids and or immunosuppressive drugs, then the rheumatologist should consider evaluation for hepatitis B, hepatitis C and tuberculosis and record the results.	Eligible Y/N	
	Passed Y/N/NA	
IF a patient is diagnosed with SLE, THEN the patient's history of vaccinations should be recorded. Patients should be vaccinated against influenza and pneumococcus if there are no contraindications.	Eligible Y/N	
	Passed on EMR Y/N/NA	
	Passed on PSR Y/N/NA	
	INFLUENZA vaccine	
	PNEUMONIA vaccine	
	DOCTOR RECOMMENDED?	
IF a patient is diagnosed with SLE, THEN the following antibodies should be evaluated at the first visit: ANA, dsDNA, Ro, La, RNP, Sm, antiphospholipid antibodies.	Eligible Y/N	
	Passed Y/N/NA	
Quality indicators - US set		
IF a patient has suspected diagnosis of SLE, THEN an initial work up should include the following: ANA, CBC, creatinine, urinalysis.	Eligible Y/N	
	Passed Y/N/NA	
IF a patient is diagnosed with SLE, the following should be ordered within 6 months of diagnosis: dsDNA, complement, antiphospholipid antibodies.	Eligible Y/N	
	Passed Y/N/NA	
If a patient has SLE, THEN education about sun avoidance should be documented at least once in the medical record.	Eligible Y/N	
	Passed on EMR Y/N/NA	
	Passed on PSR Y/N/NA	
If a patient has SLE and is on immunosuppressive therapy, THEN an inactivated influenza vaccination should be administered annually unless there is a contraindication or patient refusal is noted.	Eligible Y/N	
	Passed on EMR Y/N/NA	
	Passed on PSR Y/N/NA	
If a patient has SLE and is on immunosuppressive therapy, THEN a pneumococcal vaccination should be administered unless there is a contraindication or patient refusal is noted.	Eligible Y/N	
	Passed on EMR Y/N/NA	
	Passed on PSR Y/N/NA	
IF a patient with SLE received prednisolone $\geq$7.5mg/day for 3 months, THEN the patient should have BMD testing recorded unless patient is already on antiresorptive therapy.	Eligible Y/N	
	Passed Y/N/NA	
IF a patient with SLE received prednisolone $\geq$7.5mg/day for 3 months, THEN supplemental vitamin D and calcium should be prescribed or recommended and documented.	Eligible Y/N	
	Passed on EMR Y/N/NA	
	Passed on PSR Y/N/NA	
IF a patient with SLE received prednisolone $\geq$7.5mg/day for 3 months AND has a T score of -2.5 OR has a fragility fracture, THEN the patient should be treated with antiresorptive or anabolic agent unless contraindicated.	Eligible Y/N	
	Passed Y/N/NA	
IF a patient is prescribed a new medication for SLE, THEN a discussion with the patient about the risks versus benefits of the chosen therapy should be documented.	Eligible Y/N	
	Passed on EMR Y/N/NA	
	Passed on PSR Y/N/NA	
IF a patient is prescribed NSAID, DMARD or prednisolone, THEN baseline pathology should be documented.	Eligible Y/N	
	Passed Y/N/NA	
IF a patient is established on NSAID, DMARD or prednisolone, THEN routine pathology should be documented.	Eligible Y/N	
	Passed Y/N/NA	
IF a patient is taking prednisolone $\geq$10mg for >3 months, THEN an attempt should be made to taper the prednisolone, add a steroid sparing agent or escalate the current steroid sparing agent unless refusal or contraindications.	Eligible Y/N	
	Passed Y/N/NA	
IF a patient has evidence of SLE renal disease (proteinuria, active sediment, rising creatinine OR biopsy) in the past 2 years, THEN the following should be obtained at 3 monthly intervals: CBC, creatinine, urinalysis, urine PCR.	Eligible Y/N	
	Passed Y/N/NA	

IF a patient has been diagnosed with proliferative renal disease (class III or IV), then prednisolone and another immunosuppressant should be initiated and documented within 1 month of diagnosis, unless refusal or contraindicated.	Eligible Y/N	
	Passed Y/N/NA	
IF a patient has renal disease and proteinuria >300mg/day or eGFR <60 AND two BP readings of >130/80mmHg over 3 months, THEN patient should be treated for hypertension.	Eligible Y/N	
	Passed Y/N/NA	
IF a patient has proteinuria >300mg/day, THEN patient should be treated with ACEI or ARB unless contraindicated.	Eligible Y/N	
	Passed Y/N/NA	
IF a patient has SLE, THEN risk factors for cardiovascular disease including smoking, BP, BMI, diabetes and serum lipids should be evaluated annually. *PSR – Performed by family physician?*	Eligible Y/N	
	Passed on EMR Y/N/NA	
	Passed on PSR Y/N/NA	
IF a patient with SLE is pregnant, THEN antiphospholipid, Ro and La antibodies should be documented in the record.	Eligible Y/N	
	Passed Y/N/NA	
IF a patient has had pregnancy complications as a result of antiphospholipid syndrome, THEN patient should be offered aspirin and heparin for subsequent pregnancies.	Eligible Y/N	
	Passed Y/N/NA	
IF a woman between 18 and 45yo is started on any of the following for SLE: chloroquine, quinacrine, methotrexate, azathioprine, leflunomide, mycophenolate, cyclophosphamide or thalidomide, THEN a discussion with the patient about the potential teratogenic risks of therapy and about contraception should be documented, unless the patient is unable to conceive (e.g., hysterectomy, post-menopausal).	Eligible Y/N	
	Passed on EMR Y/N/NA	
	Passed on PSR Y/N/NA	

Abbreviations: ACE inhibitor angiotensin-converting enzyme inhibitor, ANA antinuclear antibody, anti-dsDNA anti–double-stranded DNA, ARB angiotensin receptor blocker, BMD bone mineral density, BMI body mass index, BP blood pressure, CBC complete blood cell count, DMARD disease-modifying antirheumatic drug, EMR electronic medical record review, eGFR estimated glomerular filtration rate, ESR erythrocyte sedimentation rate, EU European, ISN/RPS International Society of Nephrology/Renal Pathology Society, NSAID nonsteroidal anti-inflammatory drug, PCR protein/creatinine ratio, PSR patient self-report, SLE systemic lupus erythematosus, SLICC/ACR Systemic Lupus International Collaborating Clinics, WHO World Health Organization, US United States
