# Supplementary material for: Association of clinic setting with quality indicator performance in systemic lupus erythematosus: a cross-sectional study
Source: Arthritis Res Ther. 2022 Jun 22;24:150. doi: 10.1186/s13075-022-02823-9 (PMC9214991; doi:10.1186/s13075-022-02823-9)
Supplement: Supplementary file 3 — Additional file 3: Supplementary Table 3. Patient questionnaire. [file 13075_2022_2823_MOESM3_ESM.pdf]

ID:
Date:

Supplementary Table 3 : Patient questionnaire

Clinical profile	
Year of onset of lupus symptoms	
Year diagnosis of lupus confirmed	
Social history	
Ethnicity<br>Please tick one of these categories.	□ Caucasian<br>□ Southeast and Northeast Asian<br>□ Southern and Central Asian<br>□ Aboriginal and/or Torres Strait Islander<br>□ Sub-Saharan African<br>□ Others – please specify
Family history of first degree relative with confirmed diagnosis of lupus?	□ Yes<br>□ No
What is your highest level of education?<br>Tertiary education includes university, TAFE and other registered training organisations.	□ Lower than secondary<br>□ Secondary<br>□ Tertiary or above
What is your household income?	□ Less than $25 000 per annum<br>□ $35-70 000 per annum<br>□ $70-150 000 per annum<br>□ Over $150 000 per annum<br>□ Prefer not to answer
Smoking	□ Current smoker<br>□ Ex-smoker<br>□ Never smoked
Year quit smoking if ex-smoker	
What is your postcode?	
Do you have private hospital insurance?	□ Yes<br>□ No
Do you have private extras insurance?	□ Yes<br>□ No
New medications	
When starting a new medication, has your lupus doctor told you about the risks and the benefits of that medication?	□ Yes<br>□ No<br>□ Only sometimes
Preventative strategies	
Has your lupus doctor ever discussed the need to avoid sun and sun protection?	□ Yes<br>□ No
Has your lupus doctor advised you to take vitamin D and calcium supplementation?	□ Yes<br>□ No
Have you been taking hydroxychloroquine for more than 5 years?	□ Yes<br>□ No<br>□ I do not take hydroxychloroquine

ID:
Date:

Have you had an eye check with an eye specialist in the last 12 months?	☐ Yes<br>☐ No
Have you been taking prednisolone for more than 5 years in total?	☐ Yes<br>☐ No<br>☐ I do not take prednisolone
Do you have the yearly influenza vaccine (flu shot)?<br><br>If yes, what year was your last vaccine?	☐ Yes<br>☐ No
Have you had the pneumococcal vaccine (pneumonia vaccine) in the last 5 years?<br><br>If yes, what year was your vaccine?	☐ Yes<br>☐ No
Has your lupus doctor ever recommended that you have the above vaccines?	☐ Yes<br>☐ No
Do you have a regular GP (family doctor)?	☐ Yes<br>☐ No
How often do you see your GP?	☐ I don't see my GP<br>☐ Once a year<br>☐ Once every 6 months<br>☐ Once every 3 months<br>☐ More frequent than 3-monthly
Does your GP check your blood sugar and cholesterol level at least once a year?	☐ Yes<br>☐ No
Does your GP check your blood pressure?	☐ Yes<br>☐ No
Female participants only	
Number of pregnancies:	
If you are aged 18-45:<br>Has your lupus doctor warned you about pregnancy risks of certain lupus medications?	☐ Yes<br>☐ No
If you are aged 18-45:<br>Has your lupus doctor talked about contraception when taking certain lupus medications?	☐ Yes<br>☐ No
