# Supplementary material for: Association of clinic setting with quality indicator performance in systemic lupus erythematosus: a cross-sectional study
Source: Arthritis Res Ther. 2022 Jun 22;24:150. doi: 10.1186/s13075-022-02823-9 (PMC9214991; doi:10.1186/s13075-022-02823-9)
Supplement: Supplementary file 4 — Additional file 4: Supplementary Table 4. Multivariable regression of per-patient QI performance adjusted for sociodemographic, disease and healthcare determinants. [file 13075_2022_2823_MOESM4_ESM.pdf]

Supplementary Table 4: Multivariable regression of per-patient QI performance adjusted for sociodemographic, disease and healthcare determinants

Determinants	Simple linear regression Coefficients (95% CI)	Multiple linear regression with clinically relevant variables Coefficients (95% CI)	Multiple linear regression with statistically significant variables Coefficients (95% CI)
Clinic setting			
Lupus clinic	Ref	Ref	Ref
Public clinic	-13.2 (-16.5, -10.0)#	-13.3 (-17.8, -8.9)#	-13.7 (-16.7, -10.6)#
Private clinic	-14.8 (-18.1, -11.5)#	-11.5 (-16.4, -6.7)#	-12.1 (-15.4, -8.7)#
Age (years)	-	-0.05 (-0.2, 0.1)	-
Gender (female)	-	1.9 (-3.0, 6.8)	-
Ethnicity	-		-
Caucasian		Ref	
Asian		-0.3 (-4.3, 3.7)	
Other		-1.7 (-9.5, 6.2)	
Education	-		-
Primary		Ref	
Secondary		0.1 (-6.1, 6.3)	
Tertiary		1.3 (-4.9, 7.6)	
Income			
<35k pa		Ref	
35k- <70k pa		-0.2 (-4.5, 4.0)	
≥70k pa		-0.9 (-5.5, 3.6)	
Disease duration (years)	-	-0.3 (-0.5, -0.1)#	-0.4 (-0.5, -0.2)#
ACR criteria	-		
Malar rash		-1.0 (-4.5, 2.5)	-
Discoid rash		-1.5 (-6.8, 3.8)	-
Photosensitivity		-1.0 (-4.4, 2.4)	-
Oral ulcers		1.2 (-2.6, 4.9)	-
Arthritis		0.1 (-3.5, 3.7)	-
Serositis		-4.6 (-8.5, -0.7)*	-3.5 (-6.4, -0.7)*
Renal		3.3 (-0.5, 7.0)	5.6 (2.9, 8.4)#
Neurologic		3.8 (-1.4, 9.0)	-
Haematologic		-1.4 (-4.7, 1.9)	-
Immunologic		0.7 (-3.7, 5.0)	-
SDI score	-	0.2 (-1.0, 1.5)	-
SLEDAI score	-	0.4 (-0.2, 1.0)	-
Private hospital insurance	-	-1.3 (-4.9, 2.2)	-
Number of family physician visits pa	-		-
Annually/less		-3.1 (-12.6, 6.5)	
6 monthly		3.0 (-6.2, 12.1)	
3 monthly		-0.7 (-9.9, 8.5)	
2 monthly/more		-0.1 (-9.5, 9.2)	
Number of rheum visits pa	-	0.3 (-0.4, 1.0)	-
Model adj R^2	0.30	0.35	0.41
Model F ratio	F (2, 255) = 56.54, p <0.001	F (29, 147) = 4.27, p <0.001	F(5, 251) = 36.57, p <0.001

*p value <0.05, #p value <0.01

Abbreviations: ACR American College of Rheumatology, pa per annum, SLEDAI Systemic Lupus Erythematosus Disease Activity Index, SDI Systemic Lupus International Collaborating Clinics/ American College of Rheumatology Damage Index
