# Supplementary material for: Association of clinic setting with quality indicator performance in systemic lupus erythematosus: a cross-sectional study
Source: Arthritis Res Ther. 2022 Jun 22;24:150. doi: 10.1186/s13075-022-02823-9 (PMC9214991; doi:10.1186/s13075-022-02823-9)
Supplement: Supplementary file 5 — Additional file 5: Supplementary Table 5. Multivariable regression of per-QI performance adjusted for sociodemographic, disease and healthcare determinants. [file 13075_2022_2823_MOESM5_ESM.pdf]

Supplementary Table 5: Multivariable regression of per-QI performance adjusted for sociodemographic, disease and healthcare determinants

Determinants	Disease monitoring tests OR (95% CI)	Cardio-vascular risk factors assessment OR (95% CI)	Ophthalmic screening on hydroxychloroquine OR (95% CI)	Hepatitis and tuberculosis screening OR (95% CI)	Counselling when prescribed new meds OR (95% CI)	Influenza vaccination if on immunosuppression OR (95% CI)	Sun avoidance counselling ever OR (95%CI)	Teratogenic medication counselling in reproductive age women OR (95% CI)
Healthcare setting								
Lupus clinic	Ref	Ref	Ref	Ref	Ref	Ref	Ref	Ref
Public clinic	1.33 (0.20, 8.65)	0.18 (0.06, 0.48)#	0.53 (0.15, 1.92)	0.54 (0.16, 1.81)	0.03 (0.01, 0.12)#	0.04 (0.01, 0.22)#	0.07 (0.01, 0.75)*	0.06 (0.01, 0.44)#
Private clinic	0.06 (0.01, 0.31)#	0.16 (0.05, 0.52)#	3.15 (0.80, 12.40)	0.32 (0.06, 1.57)	0.27 (0.06, 1.14)	0.01 (0.00, 0.11)#	0.12 (0.01, 1.20)	0.15 (0.01, 1.66)
Age	0.99 (0.95, 1.04)	1.01 (0.98, 1.05)	1.04 (0.99, 1.08)	0.98 (0.94, 1.02)	0.96 (0.92, 1.01)	0.99 (0.95, 1.05)	0.98 (0.92, 1.03)	-
Gender (female)	3.49 (0.59, 20.76)	0.50 (0.16, 1.57)	1.59 (0.41, 6.15)	0.75 (0.16, 3.39)	0.59 (0.12, 2.90)	0.35 (0.06, 2.08)	0.08 (0.02, 0.38)#	-
Ethnicity								
Caucasian	Ref	Ref	Ref	Ref	Ref	Ref	Ref	Ref
Asian	2.11 (0.38, 11.76)	1.56 (0.64, 3.80)	0.89 (0.32, 2.50)	1.68 (0.50, 5.66)	0.90 (0.27, 3.06)	0.32 (0.07, 1.53)	1.50 (0.31, 7.17)	0.55 (0.10, 2.93)
Other	0.13 (0.02, 1.19)	1.04 (0.16, 6.73)	4.21 (0.15, 114.61)	2.21 (0.25, 19.45)	1.13 (0.12, 10.37)	0.18 (0.02, 1.89)	1.43 (0.14, 14.79)	0.22 (0.01, 5.53)
Education								
Primary	Ref	Ref	Ref	Ref	Ref	Ref	Ref	Ref
Secondary	0.35 (0.05, 2.62)	1.99 (0.45, 8.84)	0.63 (0.13, 3.13)	1.03 (0.17, 6.28)	1.24 (0.22, 6.84)	0.37 (0.04, 3.75)	0.83 (0.04, 18.15)	0.33 (0.01, 14.28)
Tertiary	1.70 (0.21, 13.92)	0.63 (0.14, 2.82)	1.36 (0.28, 6.65)	4.55 (0.75, 27.55)	2.38 (0.43, 13.09)	0.82 (0.09, 7.58)	4.33 (0.23, 81.97)	1.84 (0.05, 64.49)
Income								
<35k pa	Ref	Ref	Ref	Ref	Ref	Ref	Ref	Ref
35k- <70k pa	0.68 (0.16, 2.82)	1.73 (0.65, 4.64)	0.92 (0.28, 3.01)	0.37 (0.11, 1.30)	0.59 (0.17, 2.03)	0.59 (0.14, 2.53)	0.55 (0.11, 2.73)	0.72 (0.10, 5.14)
>=70k pa	0.51 (0.10, 2.56)	1.44 (0.49, 4.22)	0.57 (0.15, 2.15)	0.58 (0.14, 2.35)	2.21 (0.54, 9.03)	0.56 (0.12, 2.61)	0.21 (0.03, 1.52)	1.07 (0.15, 7.80)
Disease duration	0.94 (0.88, 1.01)	0.98 (0.94, 1.03)	1.01 (0.95, 1.06)	0.95 (0.89, 1.01)	1.02 (0.96, 1.08)	1.04 (0.97, 1.12)	0.92 (0.82, 1.04)	0.99 (0.87, 1.13)
ACR[a]								
Malar rash	0.59 (0.17, 2.06)	0.69 (0.30, 1.55)	0.66 (0.25, 1.75)	1.70 (0.56, 5.21)	1.69 (0.55, 5.25)	1.15 (0.33, 3.99)	0.50 (0.11, 2.27)	1.66 (0.37, 7.43)
Discoid rash	1.76 (0.28, 10.97)	1.55 (0.48, 5.06)	0.72 (0.15, 3.39)	0.37 (0.08, 1.73)	0.67 (0.13, 3.54)	0.38 (0.06, 2.54)	1.63 (0.21, 12.64)	11.64 (1.48, 91.76)*
Photo-sensitivity	0.55 (0.17, 1.76)	0.87 (0.39, 1.90)	0.52 (0.20, 1.35)	0.90 (0.33, 2.42)	1.93 (0.70, 5.37)	0.80 (0.25, 2.52)	2.89 (0.75, 11.08)	2.49 (0.51, 12.21)
Oral ulcers	1.28 (0.33, 4.96)	1.40 (0.60, 3.28)	3.23 (1.04, 10.05)*	1.03 (0.32, 3.30)	1.54 (0.49, 4.81)	1.40 (0.39, 5.08)	0.58 (0.12, 2.92)	0.43 (0.08, 2.32)
Arthritis	1.00 (0.28, 3.62)	0.85 (0.36, 2.01)	0.84 (0.29, 2.40)	1.38 (0.40, 4.73)	0.41 (0.13, 1.26)	0.34 (0.09, 1.28)	1.26 (0.29, 5.56)	0.81 (0.16, 4.21)
Serositis	0.85 (0.20, 3.52)	1.25 (0.51, 3.03)	0.47 (0.16, 1.35)	0.79 (0.26, 2.42)	0.75 (0.21, 2.60)	1.16 (0.27, 4.93)	0.44 (0.09, 2.15)	1.06 (0.15, 7.28)
Renal	2.59 (0.59, 11.36)	1.40 (0.60, 3.24)	2.43 (0.87, 6.80)	0.68 (0.23, 2.02)	3.20 (0.90, 11.41)	0.42 (0.11, 1.51)	0.39 (0.09, 1.76)	1.92 (0.33, 11.28)
Neurologic	1.07 (0.16, 7.36)	1.17 (0.33, 4.20)	0.37 (0.10, 1.41)	1.29 (0.20, 8.39)	2.60 (0.49, 13.80)	4.47 (0.59, 33.59)	2.84 (0.26, 31.54)	0.69 (0.07, 7.18)
Haematologic	0.46 (0.14, 1.48)	1.58 (0.72, 3.46)	1.12 (0.44, 2.80)	0.52 (0.19, 1.37)	1.75 (0.63, 4.90)	0.98 (0.33, 2.97)	1.76 (0.45, 6.83)	1.34 (0.26, 7.02)
Immunologic	1.93 (0.42, 8.77)	0.64 (0.22, 1.85)	1.70 (0.42, 6.81)	1.59 (0.41, 6.18)	1.36 (0.36, 5.05)	1.95 (0.43, 8.93)	0.21 (0.04, 1.15)	3.45 (0.30, 39.24)
SDI	1.21 (0.75, 1.98)	0.96 (0.72, 1.28)	0.97 (0.71, 1.33)	1.16 (0.82, 1.64)	0.82 (0.56, 1.21)	1.25 (0.85, 1.86)	1.11 (0.66, 1.87)	0.38 (0.17, 0.88)*
SLEDAI	1.23 (0.91, 1.66)	0.97 (0.84, 1.13)	1.03 (0.88, 1.21)	1.06 (0.88, 1.27)	0.86 (0.71, 1.03)	0.99 (0.81, 1.21)	1.08 (0.85, 1.37)	1.10 (0.82, 1.48)
Hospital insurance	1.24 (0.35, 4.35)	0.87 (0.38, 2.01)	1.83 (0.69, 4.84)	0.66 (0.21, 2.03)	0.79 ((0.27, 2.31)	0.95 (0.28, 3.19)	0.45 (0.10, 2.03)	0.64 (0.12, 3.48)
Regular FP	0.58 (0.03, 13.21)	12.45 (1.10, 140.95)*	-	0.71 (0.05, 9.17)	-	0.71 (0.03, 14.37)	2.93 (0.06, 141.42)	-
No of rheum visits per year	0.94 (0.71, 1.25)	1.15 (0.96, 1.38)	1.09 (0.89, 1.34)	0.90 (0.74, 1.09)	0.90 (0.72, 1.12)	1.00 (0.81, 1.23)	0.77 (0.55, 1.07)	0.87 (0.59, 1.26)

*p<0.05 #p<0.01

a: ACR criteria included to demonstrate SLE phenotype on an 'ever-present' basis.

Note: Quality indicators for which performance was high globally or observation numbers were small were excluded from multivariable regression analyses.

Abbreviations: ACR American College of Rheumatology, FP family physician, SLEDAI Systemic Lupus Erythematosus Disease Activity Index, SDI Systemic Lupus International Collaborating Clinics/ American College of Rheumatology Damage Index
