# Supplementary material for: Association of clinic setting with quality indicator performance in systemic lupus erythematosus: a cross-sectional study
Source: Arthritis Res Ther. 2022 Jun 22;24:150. doi: 10.1186/s13075-022-02823-9 (PMC9214991; doi:10.1186/s13075-022-02823-9)

Supplementary Figure 1: Per-patient QI performance multivariable linear regression model residual versus fit plot

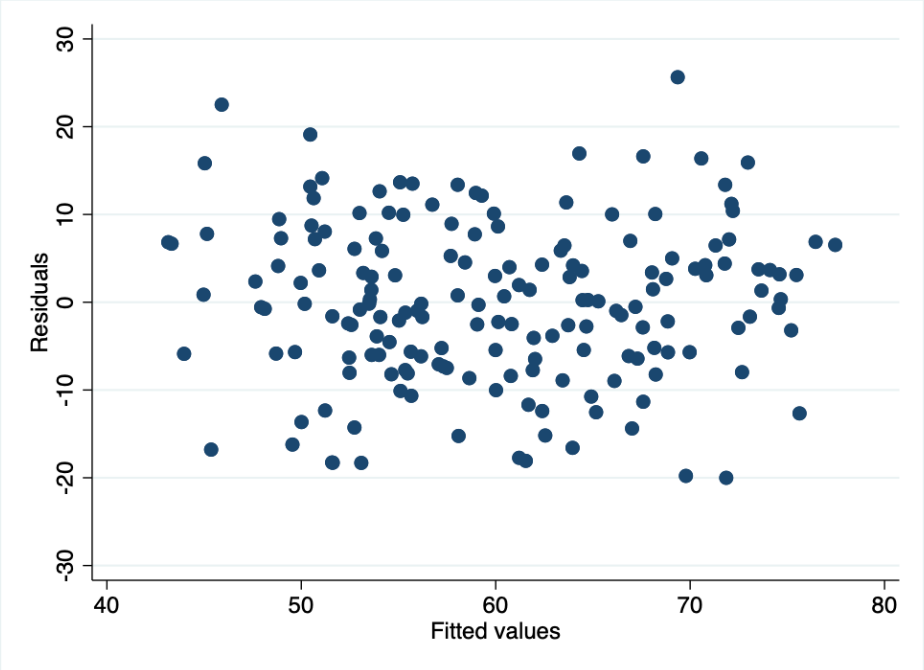

Supplement: Supplementary file 6 — Additional file 6: Supplementary Figure 1. Per-patient QI performance linear multivariable regression model residual versus fit plot. [file 13075_2022_2823_MOESM6_ESM.pdf]
